# An Outline of Data Aggregation Security in Heterogeneous Wireless Sensor Networks

**DOI:** 10.3390/s16040525

**Published:** 2016-04-12

**Authors:** Sabrina Boubiche, Djallel Eddine Boubiche, Azzedine Bilami, Homero Toral-Cruz

**Affiliations:** 1 LaSTIC Laboratory, Department of Computer Science, University of Batna, Batna 05000, Algeria; dj.boubiche@univ-batna (D.E.B.); bilamiaz@univ-batna (A.B.); 2 Department of Sciences and Engineering, University of Quintana Roo, Chetumal 77019, Mexico; htoral@uqroo.edu.mx

**Keywords:** data aggregation, wireless sensor network, heterogeneity, security

## Abstract

Data aggregation processes aim to reduce the amount of exchanged data in wireless sensor networks and consequently minimize the packet overhead and optimize energy efficiency. Securing the data aggregation process is a real challenge since the aggregation nodes must access the relayed data to apply the aggregation functions. The data aggregation security problem has been widely addressed in classical homogeneous wireless sensor networks, however, most of the proposed security protocols cannot guarantee a high level of security since the sensor node resources are limited. Heterogeneous wireless sensor networks have recently emerged as a new wireless sensor network category which expands the sensor nodes’ resources and capabilities. These new kinds of WSNs have opened new research opportunities where security represents a most attractive area. Indeed, robust and high security level algorithms can be used to secure the data aggregation at the heterogeneous aggregation nodes which is impossible in classical homogeneous WSNs. Contrary to the homogeneous sensor networks, the data aggregation security problem is still not sufficiently covered and the proposed data aggregation security protocols are numberless. To address this recent research area, this paper describes the data aggregation security problem in heterogeneous wireless sensor networks and surveys a few proposed security protocols. A classification and evaluation of the existing protocols is also introduced based on the adopted data aggregation security approach.

## 1. Introduction

Data aggregation is an important basic concept in wireless sensor networks (WSNs), which has been proposed to optimize the data collection process and the sensor nodes’ energy reserves. Indeed and due to the network density, the data collection process suffers from redundancy and data interrelation which may drain the sensor node batteries and affect the network performance (overhead, transmission bandwidth, latency…). Thus, it is necessary to use methods for merging data at the intermediate nodes in order to reduce the number of packets transmitted to the base station, thus allowing energy and bandwidth savings. This can be accomplished through a data aggregation mechanism.

Despite data aggregation advantages it can lead to some security failings: Usurpation of identity, deliberate removal of the packets, alteration of sensory readings and aggregation results. Many security solutions have been proposed to secure the data aggregation process. These solutions are mainly proposed for homogeneous WSNs where nodes are identical in capacity, energy consumption and hardware complexity. The proposed solutions aim to guarantee secure data aggregation and an authenticated broadcast based on a variety of security mechanisms like symmetric encryption [1] and authentication message MAC [2]. Most of these mechanisms are simple and can only guarantee a minimum security level. Also, they cannot tolerate the use of high security level and more complex algorithms, like asymmetric encryption [1] and privacy homomorphism [3], which require additional computing power, energy reserves and memory. Indeed, simple sensor nodes cannot support complex and powerful mechanisms.

**Figure 1:**
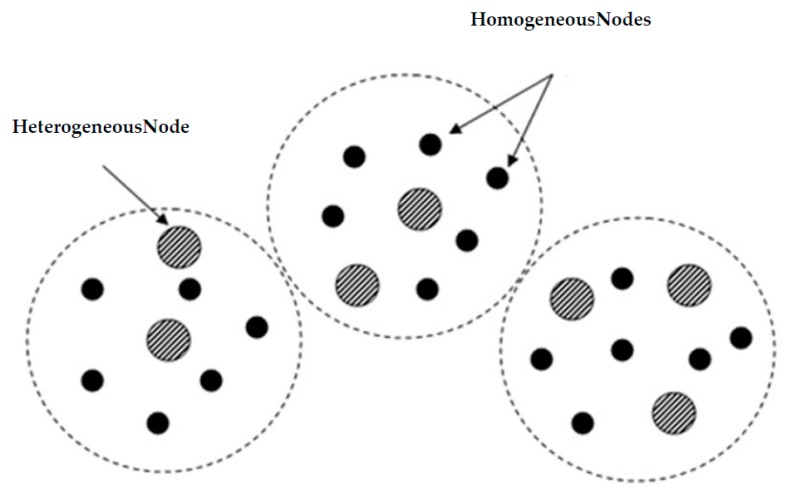
Heterogeneous Wireless Sensor Network.

Heterogeneous WSNs represent a new class of technology and a recent research area that overcomes the limits of WSNs, especially in the security field. The continued progress in wireless sensor networks, especially in the miniaturization of the processors has enabled the development of several varieties of nodes. When more than one type of nodes is used in a WSN, it is called heterogeneous [4]. A typical heterogeneous wireless sensor network is composed of a large number of homogeneous nodes and a reduced number of heterogeneous nodes as shown in Figure 1. Identical nodes, which principally collect data, are inexpensive (limited resources) while the heterogeneous nodes are more expensive, dispose of high computing and resource capacity (memory and energy reserves). Indeed, deploying heterogeneous high capacity sensor nodes in a network contributes to the efficient distribution of the workload between the network nodes. Thereby, the high resource capacity sensor nodes are charged with accomplishing complex and high energy consumption tasks while simple and low energy consumption tasks are delegated to the resource limited sensor nodes.

The resource capacities of the heterogeneous sensor nodes have opened new security research directions since complex and high security level algorithms can be used to secure the data aggregation process. Several security techniques are used to secure the data aggregation process in heterogeneous WSNs, the base of which is represented by the privacy homomorphism algorithm [3]. The main idea is to apply the aggregation functions directly to the encrypted data, thus protecting the data integrity while transmitting them from the aggregation nodes to the base station. The data aggregation security problem in heterogeneous WSNs is relatively new and the proposed security protocols are numberless compared to the homogeneous ones. In addition to the main security issues, a heterogeneous data aggregation security protocol must guarantee the following points:

- Exploitation of the high capacities of the heterogeneous sensor nodes.
- Respect of the resources constraints of the simple homogeneous sensor nodes.
- Optimization of the data aggregation process while ensuring high data accuracy.

The main contribution of this paper is to survey the existing security aggregation protocols dedicated to the heterogeneous WSNs. In our opinion, the heterogeneous data aggregation security protocols should be identified and differentiated from those dedicated to the homogeneous WSNs, since they cannot be applied to this classical type of networks. On the other hand, adopting homogeneous data aggregation security protocols in heterogeneous WSNs may underexploit the network resources capacities. To the best of our knowledge, only eight protocols have been proposed and we think that it is interesting to shed light on these protocols and discuss their security strategies. The rest of the paper is organized as follows: Section 2 presents a classification and a survey of the different published heterogeneous data aggregation security protocols. In Section 3, we evaluate and discuss the surveyed heterogeneous data aggregation security protocols based on the adopted security approach. Finally, we conclude the paper in Section 4.

## 2. Data Aggregation Security Mechanisms for Heterogeneous Wireless Sensor Networks

**Figure 2:**
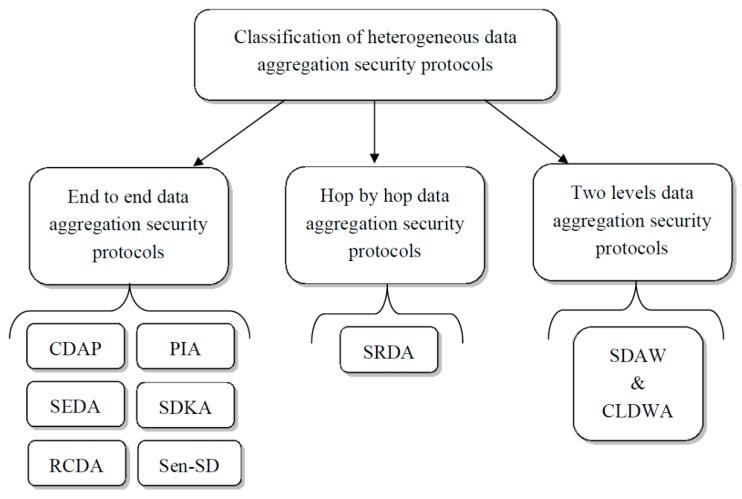
Heterogeneous data aggregation security protocol classification.

Secure data aggregation protocols for heterogeneous WSNs can be classified into two types: End to end and hop by hop data aggregation protocols [5,6]. In end to end data aggregation protocols, security is guaranteed by applying the aggregation function directly to the encrypted data. In the second type of protocols, data must be decrypted at each aggregation node, thereby losing the end to end privacy between the transmitter and the receiver. Recently a third type of data aggregation security protocol has been introduced, which divides the network into two stages: High resource capacity heterogeneous nodes and simple, limited resource capacity sensor nodes. This new type of protocol introduces a two level security strategy where each network stage applies different security approaches. The heterogeneous data aggregation security protocol classification is presented in Figure 2.

### 2.1. End to End Secure Data Aggregation Protocols

**Figure 3:**
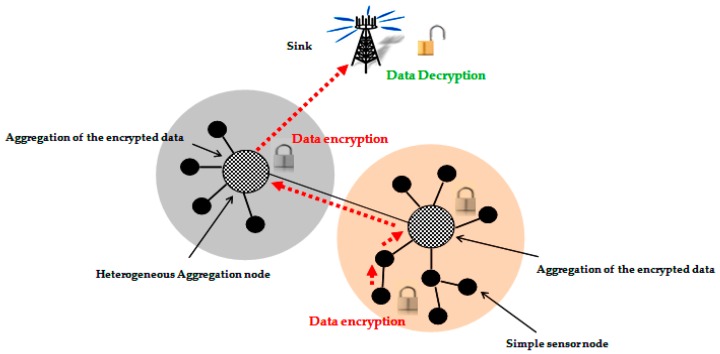
End to end data aggregation security approach.

End to end secure data aggregation protocols aim to insure data privacy and confidentiality from the sensor nodes to the base station (Figure 3). To offer more security, sensed data is concealed and the nodes perform the aggregation functions directly on the encrypted data. Most of the end to end secure data aggregation protocols use privacy homomorphism, which assures this type of security.

#### 2.1.1. Concealed Data Aggregation in Heterogeneous Sensor Networks Using Privacy Homomorphism (CDAP)

Ozdemir [7] has proposed the CDAP protocol to facilitate the aggregation process and to obtain a safe end to end transmission between the base station and the nodes of the network. Therefore, he proposed the use of privacy homomorphism to provide end to end data concealment, and also to operate directly on cipher texts while providing a secure data aggregation process. The protocol is applied to a heterogeneous WSN where powerful nodes called AGGNODEs are utilized.

##### Protocol Presentation

CDAP exploits privacy homomorphism [3] to insure end to end confidentiality and a secure data aggregation process. Privacy homomorphism is an encryption transformation which allows direct calculation on encrypted data. It can be based on symmetric or asymmetric encryption. However, the use of symmetric key leads to selected text attack vulnerability. Thus, according to the author, the use of privacy homomorphism based on asymmetric keys is preferred. To support the high calculation costs of the encryption based on asymmetric cryptographic privacy homomorphism, the protocol uses a set of nodes rich in resources called AGGNODEs; where the privacy homomorphic encryption occurs. CDAP is divided into the following phases:

- The public key of the base station is assigned to the AGGNODEs and the network is deployed.
- Pair-wise keys are shared between the AGGNODEs and their neighbouring nodes.
- Each node encrypts data using the symmetric encryption algorithm (RC5) [8] and sends the encrypted data to its neighbor AGGNODE.
- Each AGGNODE decrypts the received data, applies the aggregation function and encrypts the result using the privacy homomorphism encryption algorithm described as follows: Let *E* be the encryption function and *D* the decryption function. + and * indicate the addition and the multiplication functions applied on the data set *Q*. Assuming that the private and the public keys of the base station are respectively $K_{pr}\;\;$and $K_{pu}$, a transformation encryption is additively homomorphic if:
  (1) $$a+b=\left(DK_{pr}\left(EK_{pu}\left(a\right)+EK_{pu}\left(b\right)\right)\right)$$
  where *a*, *b* belong to *Q*. This transformation is multiplicatively homomorphic if:
  (2) $$a*b=\left(DK_{pr}\left(EK_{pu}\left(a\right)*EK_{pu}\left(b\right)\right)\right)$$
- The encrypted data are transmitted and the AGGNODEs aggregate hierarchically the encrypted data during the transmission.
- Only the base station can decrypt the aggregated data using its private key.

##### Evaluation

CDAP is dedicated to heterogeneous WSNs. CDAP increases the capacity of securing the data aggregation mechanism when compromised nodes are detected. The network performance using AGGNODEs is evaluated compared to the homogeneous WSNs, where only the base station has higher calculation capacities and larger storage space. Thus, through the AGGNODEs and data aggregation, energy efficiency and the bandwidth are improved while ensuring secure communication. The performance evaluation shows that the protocol could be applied to large heterogeneous WSNs.

However, in spite of the increased degree of security, the use of the privacy homomorphism faces limits, including the restricted number of allowed aggregation functions. In addition, the author did not give any indications about the key generation and distribution mechanisms.

We assume that since privacy homomorphism is applied on the AGGNODEs, their high capacities are largely exploited. However, due to the use of symmetric encryption at the homogeneous nodes, the data aggregation accuracy can be affected by the latency introduced during the encryption phase. Also, the homogeneous simple sensor nodes have limited capacities and the use of symmetric encryption may not be tolerable by these nodes.

#### 2.1.2. Privacy-Preserving Integrity-Assured Data Aggregation (PIA)

Taban *et al.* [9] have addressed the problem of data aggregation, with the guarantee of data integrity, and private information efficiency and protection as a common goal. In their model, the aggregation node is used as an intermediate between the user and the sensor nodes. The problem is that the user wants to check the integrity of the received aggregated data while the owner of the network prefers maintaining them secret from the user. In this context, the authors propose four solutions.

##### Protocol Presentation

- The first solution uses the privacy homomorphism encryption to conceal data. The homomorphism combined with authentication message MAC are used to construct an authenticated encryption protocol for the aggregation model [10]. However, the homomorphism allows a limited number of aggregation functions such as sum or average.
- The second solution uses the Order Preserving Encryption Scheme or OPES [11] which maintains the data fusion confidentiality. As an example, OPES preserves the encrypted order so that any data pair $y_{1}$ and $y_{2}$ where $y_{1}<y_{2}$ are encrypted to $c_{1}$ and $c_{\;2\;}$ so that$\;\;c_{1}<c_{2}$. However, this scheme is only required for the aggregation functions which can be approximated with uniform sampling. It is also exclusively based on the comparison operation.
- The third solution uses the Secure Hierarchical In-networking Aggregation (SHIA) protocol [12], which supports all the possible aggregation functions to adapt a distributed integrity. However, the communication cost that corresponds to this protocol is O(N) messages per sensor node where *N* is the total number of the nodes in the network.
- To insure data confidentiality and integrity, the third solution is improved by introducing a logical aggregation tree in the aggregation node. This solution is the latest one in which the communication cost is O(logN). However, this solution supports only a limited number of aggregation functions such as average, and Min/Max.

##### Evaluation

The PIA protocol ensures end to end security and exploits the high capacity of heterogeneous sensor nodes, however, the authors did not indicate how the heterogeneous nodes are deployed. In addition, the heterogeneity exploitation is not sufficiently detailed. The use of homomorphic encryption on aggregation nodes affects significantly the data aggregation process as complex data aggregation functions cannot be applied. The proposed solutions to overcome the restriction of the data aggregation functions (SHIA *(Secure Hierarchical In-networking Aggregation)* and OPES *(Order Preserving Encryption Scheme)*) may reduce the data aggregation accuracy due to the introduced computation delay and may drain the resource capacities of the homogeneous simple sensor nodes.

#### 2.1.3. Secure-Encrypted Data Aggregation for Wireless Sensor Networks (SEDA)

Huang *et al.* [13] have proposed this protocol to eliminate redundant sensor readings without encryption and to maintain data confidentiality during their transmission. The proposed protocol insures data security and confidentiality. Redundant sensed data is aggregated into a single package. The protocol is dedicated to centralized WSNs where the network is divided into groups of nodes [14]. In each group, the leader is responsible of performing the aggregation function. The leaders have more powerful radio antennas to transmit directly to the base station. The authors assume that the sensor nodes can transmit only to the aggregation nodes, consequently reducing the transmission costs and the energy power. The authors propose an aggregation mechanism to maintain data confidentiality and secrecy; the sensor nodes encrypt data before transmitting them to the aggregation nodes. Data thus remains secret from the aggregation node. In addition, the protocol is effective against known-plaintext attacks, selected text attacks and encrypted attacks.

##### Protocol Presentation

The protocol is divided into two phases: The encryption phase and the aggregation phase:

- *Encryption phase*: The encryption phase insures data confidentiality with lower costs. During this phase, each sensor node randomly generates a new key, and changes periodically the encryption keys.
- *Aggregation phase:* The aggregation mechanism enables the identification of two identical readings. All redundant readings of *n* encrypted packets are thus eliminated. If *n* identical readings are encrypted and transmitted to the aggregation node, the aggregation node must verify these entries n−1 times and save n−1 packet transmissions.

##### Evaluation

Due to the use of more powerful aggregation nodes, we suppose that this protocol is dedicated to heterogeneous WSNs. In this protocol, the aggregation nodes do not decrypt the received data before applying the aggregation function, thus saving more energy. Data is encrypted using a XOR *(exclusive OR logical operator)* operator and a hash function. The encryption key, and consequently the sensed data, are hidden from the aggregation nodes. With the use of random keys to encrypt the sensed data, the protocol becomes more robust against attacks. Also, the random generation of the new keys by each node offers more privacy.

According to the heterogeneity requirements, we consider that the use of heterogeneous nodes is not optimal and that the higher capacities of the aggregation nodes are not exploited through the use of powerful and complex security primitives. Indeed, the encryption is not performed by the heterogeneous nodes. Also, the homogeneous simple sensor nodes encrypt data and generate new keys periodically, thus exceeding their limited capacities. In another other side, data aggregation accuracy can be significately reduced through the use of the symmetric encryption on the simple sensor nodes (high computation delay).

#### 2.1.4. Secure Aggregation with Key Management (SDKAM)

SDKAM was proposed by Sandhya and Murugan [15]. Its main objective is to provide a secure environment for heterogeneous WSNs using the privacy homomorphism protocol. SDKAM exploits the heterogeneity of the sensor nodes to treat the encrypted data. It also provides an effective key management system for data communication between the sensor nodes, and uses more powerful aggregation nodes which must be able to perform the aggregation functions directly on the encrypted data. To provide additional security, the authors assume that an effective key management system for the communication between the sensor nodes must be used. In SDAKM, the authors insure a secure data aggregation using Hill Cipher (IHC) iteration technique [16]. IHC is an additive privacy homomorphism system for communication between the aggregation nodes. To insure a safe communication between the sensor nodes in the network, an efficient key management is presented. It is based on key pre-distribution and revocation, and on a decentralized protocol for key management. It exploits the sensor nodes heterogeneity compared to the computing capabilities. The problem of the revoking keys in the compromised nodes is resolved through a distributed protocol.

##### Protocol Presentation

IHC encryption and decryption mechanisms are organized as follows:

(1) Encryption Mechanism

The encryption function is given by: E(*):VI→VIxVI and the encryption process is given as follows:

- A random encryption matrix *A* Є Ml × l and an initialization vector u Є *Vl* are chosen.
- Set u=x−1 and$\;x_{0}=m$, where *m* is the vector *Vl* Є unencrypted data.
- For *0 ≤ i <k*, calculate $A_{xi}=x_{i+1}-x_{i-1}$
- The encrypted data is represented by: $c=\left(x_{k}\;,\;x_{k-1}\right)$

(2) Decryption Mechanism

With $c=\left(x_{k}\;,\;x_{k-1}\right)$ as an initial condition, iterate *k* times the following to return to $x_{0}$ which is unencrypted data: $A_{xi}=x_{i-1}-x_{i+1}$. The main advantage of this iterative algorithm is that there is no restriction on the choice of *A*.

- *Effective key management system:* The authors propose an efficient key management in heterogeneous WSNs. This system is based on a decentralized algorithm for key pre-distribution and revocation, which includes the following phases:
- *Key Pre-distribution:* In this phase, the keys are chosen from pools of keys and then pre-distributed.
- *Shared key discovery phase:* A common key between the neighboring nodes is chosen among the pre-distributed keys.
- *Key path establishment phase:* After the secret key is revealed, a secure data transmission path, including encryption and decryption, is established.
- *Key revocation phase:* If a node is compromised, the shared keys are revoked through a voting algorithm. Compromised nodes are identified according to their behavior, and are isolated.

##### Evaluation

The SDAKM protocol is dedicated to heterogeneous WSNs. SDAKM ensures a secure data aggregation and can significantly increase the security degree of the network by providing a secure path through the data encryption process, and an efficient and decentralized key management protocol. Indeed, to decrypt an IHC encrypted text, the matrix *A* of secret keys and the number of iterations *k* must be known.

Therefore, even if an intruder knows the matrix of the keys *A*, he cannot decrypt any given cipher text without having a knowledge about *k*. Therefore, if the number of iterations *k* is kept secret as a part of the private key, IHC security is increased. In addition, if another vector *u* is used to encrypt each pure data vector, IHC security could be significantly improved.

The main requirement expected while deploying heterogeneous nodes is to exploit their high capacities of computation. SDKAM is based on the use of the privacy homomorphism and the symmetric keys, thus reinforcing security while efficiently exploiting the high capacities of the heterogeneous nodes. However, the protocol faces the limited number of allowed aggregation functions which reduce the degree of data aggregation. We assume that the accuracy requirement is not satisfied due to the use of the symmetric encryption which also affects the limited capacities of the homogeneous simple sensor nodes.

#### 2.1.5. Recoverable Concealed Data Aggregation for Data Integrity in Wireless Sensor Networks (RCDA)

RCDA was proposed by Chen *et al.* [17]. To confirm data integrity and authenticity, the base station can recover all the sensed data even if they are aggregated (in the other protocols, the base station cannot recover the aggregated data). In addition, the protocol is adopted for both homogeneous and heterogeneous WSNs. RCDA offers two algorithms dedicated to homogeneous and heterogeneous WSNs, namely RCDA-HOMO and RCDA-HETE. In this study, we focus on RCDA-HETE. In RCDA-HETE, there are two types of nodes: the low end (L)-nodes represent the majority of the network nodes. The L-nodes have a low capacity. The second type of nodes are the high end (H)-node. The H-nodes are more powerful nodes, they can store more keys if necessary.

##### Protocol Presentation

RCDA-HETE is divided into five procedures: installation, intra-group encryption, intergroup encryption, aggregation and verification. During the installation procedure, necessary keys are loaded in the L-nodes and the H-nodes. Each L-node must share a key with the group leader. If the base station knows the information about the group before the deployment, the keys can be pre-loaded in all the L-nodes and the H-nodes. However, in the majority of WSNs, the nodes are deployed randomly.

The encryption process begins when the L-nodes of the intra-group want to send their collected data to the corresponding H-node. The objective of this process is to establish a secure channel between the nodes. For example, a node $L_{i}\;$encrypts data with key $K_{i}$ and sends it to node $H_{1},\;$which proceeds to the decryption.

In the intergroup encryption procedure, each H-node aggregates the received data. Then, the H-node encrypts and signs the obtained result. In addition, if the H-node receives the encrypted data and the signatures from the other nodes in the routing path, it will activate the aggregation function.

Finally, the verification procedure realized by the base station insures the integrity and the authentication of the aggregation results.

##### Evaluation

RCDA-HETE is a heterogeneous protocol which insures data integrity and authentication. Indeed, RCDA-HETE allows the verification of each sensed data using the H-nodes. The intergroup encryption allows the L-nodes to send encrypted collected data and an authentication message MAC to their leader to verify the data integrity. RCDA-HETE can also use diverse aggregation functions pre-loaded in the H-nodes before the deployment of the network. Thus, the nodes can execute the aggregation functions according to the application needs.

RCDA-HETE is secured against malicious attacks. Indeed, the intra-group traffic is encrypted using a pair-wise key and the protocol generates a signature corresponding to each received data. Therefore, the attacker nodes cannot modify the messages or inject false data without the private key. If a malicious node compromises the sensor nodes, the following situations are considered: The adversary can compromise a sensor node and use it as a legal node. Additionally, if the value of a message is altered within a reasonable range, its detection becomes difficult. A malicious node can also try to alter the aggregation result. It can also impersonate the other nodes. The proposed protocol provides security against these attacks using a signature for each generated message. However, we consider that due to the key pre-distribution, the network becomes not scalable. Also, the key management system was not given by the authors.

As RCDA-HETE is only based on the use of the simple symmetric encryption between the sensor nodes and the aggregation nodes, we assume that the first data aggregation security requirement in the heterogeneous WSN which is the high exploitation of the heterogeneous nodes capacities is not satisfied.

#### 2.1.6. Recoverable Concealed Data Aggregation for Data Integrity in Wireless Sensor Networks (Sen-SDA)

Sen-SDA was proposed by Shim [18]. Sen-SDA is a secure data aggregation mechanism based on the combination of the homomorphic encryption scheme (HE), EC-El Gamal^+^, an identity based signature scheme (IBS), and a batch verification technique with binary quick search (BQS) for finding invalid signatures. The used cryptographic primitives are adapted to heterogeneous clustered WSNs. Additive HE scheme is used to reduce the total length of the ciphertexts and to achieve end-to-end confidentiality. Due to the (IBS) scheme, hop-by-hop authentication is insured by verifying all the transmitted encrypted data. The batch verification technique is used to reduce the verification costs for multiple signatures. The invalid signatures in the batch verification are identified through the BQS using efficient methods [19].

In their network model, the authors have assumed a WSN consisting of one stationary fixed BS which serves as a private key generator (PKG). The BS is responsible for the generation of the private keys for all the nodes. The network consists also of a large number of stationary heterogeneous sensor nodes with unique identifiers (IDs). Each node in the network is mapped to exactly one cluster and can directly communicate with its CH which verifies all the signatures received from its member nodes, applies the aggregation function to the ciphertexts, signs the aggregated ciphertext and sends the result directly to the BS.

##### Protocol Presentation

- The BS generates the system parameter which is preloaded in each sensor node.
- The BS keeps a master secret key for the extraction of the private key and a secret key for the decryption of the ciphertexts.

To send a ciphertext-signature pair, each sensor node performs the following steps:

- Each sensor node encrypts a message using the public key of the BS and obtains a ciphertext through EC-ElGamal^+^.
- The node uses then the private key to generate a signature.

After receiving the signed messages from its member nodes, each CH verifies the validity of the timestamps before performing the following steps:

- The CH uses the batch verification technique to find and reject the invalid signatures.
- The CH then aggregates the received messages with the private key, generates a new signature and sends the signed messages to the BS.

The BS verifies the validity of the timestamps before performing the following steps:

- The BS uses the batch verification technique with BQS until the batch verification is completed successfully.
- If the batch verification is completed successfully, the BS recovers the message using the secret key corresponding to the public key.

##### Evaluation

Due to the use of homomorphic encryption, Sen-SDA is a secure protocol and ensures end-to-end confidentiality since the data is encrypted from the source to the destination using asymmetric encryption. To insure a higher degree of security and to overcome the insecurity of the homomorphic schemes against message modification attacks and injection attacks, the proposed homomorphic encryption is combined to a signature scheme.

Sen-SDA also guarantees hop by hop authentication. Indeed, in Sen-SDA, all the intermediate CHs sign their aggregated data after verifying the signatures received from member nodes, and during the signatures verification, the CHs can filter the invalid signatures.

To overcome the computational cost of public-key certificate distributions, verifications and storage Sen-SDA is based on the use of an efficient pairing free identity-based signature scheme.

We assume that the combination of the homomorphic encryption scheme (HE), EC-El Gamal^+^, an identity based signature scheme (IBS), and a batch verification technique with binary quick search (BQS) for finding invalid signatures is a good way to exploit the heterogeneous nodes’ high capacities. However, the use of asymmetric encryption on simple heterogeneous nodes is not optimal due to the limited capacities of the sensor nodes. Also, homomorphic encryption significantly reduces the use of the aggregation functions which affects the data aggregation process and reduces the data aggregation accuracy.

### 2.2. Hop by Hop Secure Data Aggregation Protocols

**Figure 4:**
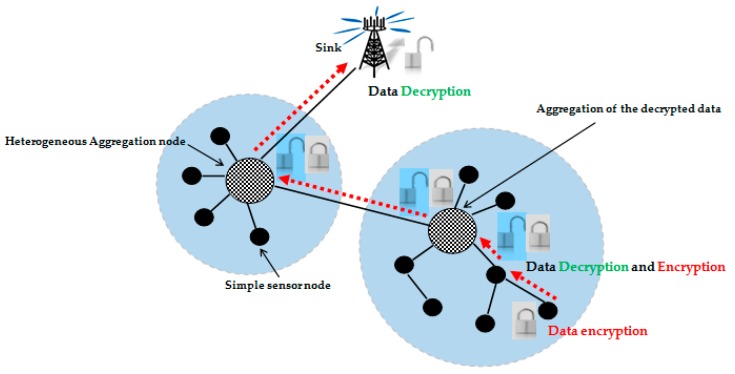
Hop by hop data aggregation security approach.

Hop by hop secure data aggregation protocols aim to provide strong data aggregation while ensuring data integrity (Figure 4). Nevertheless, decrypting data at each aggregation node may affect their confidentiality. Data is thus more vulnerable to malicious attacks once the encryption key is discovered. For this reason, these protocols are not largely used, particularly when security is related to sensitive application areas. In heterogeneous WSNs and according to our research, only one hop by hop secure data aggregation protocol was developed.

#### 2.2.1. Secure Reference-Based Data Aggregation Protocol for Wireless Sensor Networks (SRDA)

Sanli *et al.* [20] have proposed a hop by hop secure data aggregation system based reference which applies different security degrees at different levels of the hierarchy (aggregation nodes). According to the authors, the degree of security should be increased gradually as the messages are transmitted through the levels. They opted for a cryptographic algorithm which adjusts its parameters and the number of encryption rounds to modify the degree of security.

The basic idea of SRDA is that the nodes will transmit reference data instead of pure sensed data and only the difference between them is used. SRDA first provides a key distribution protocol with low memory overhead to secure the communication links, and then, to save energy, it uses a security mechanism with a variant strength. In this protocol, differential encrypted data representing the difference between the reference value and the sensed data are transmitted to the aggregation nodes instead of the pure sensed data, to reduce the number of transmitted bits. The reference value is the average of *N* readings.

##### Protocol Presentation

To guarantee security, secret keys are shared between the communicating nodes. To reduce the overload in the key distribution system, additional information such as the location of the sensor nodes can be used. After the network is deployed, the nodes begin an installation key phase or a shared key discovery phase. The aggregation protocol SRDA starts next. Its main steps are summarized as follows:

- A node, which can be a sensor node or an aggregation node, transmits the results to a higher level, forwards the packet containing the pure sensed data $M_{t}$ at time *t* for the first packet in the session.
- The group leader creates a reference input $M_{t}$ for this node.
- For subsequent readings M(t+j), *j* > 1, the sensor node sends differential data $\left(M\left(t+j\right)-M_{t}\right)$ instead of the pure sensed data M(t+j).
- When the session is finished, the group leader removes the reference input of the node.

After receiving the sensed data, the aggregation nodes proceed as follows:

- Decrypt data and calculate the number of hops to the base station.
- Execute the aggregation function, and then encrypt the obtained result using RC6 algorithm (block cipher) [21]. The number of hops *NS* is calculated as follows: $NS=\frac{1}{hops}*100$
- Transmit the encrypted result to the base station.
- The base station decrypts the received data using the corresponding decryption key.

##### Evaluation

SRDA protocol is proposed to guarantee data confidentiality using RC6 encryption algorithm. It also ensures data freshness by combining the key and the data aggregation updating. SRDA reduces the number of transmitted bits and increases the network security. It also reduces the energy consumption and bandwidth by sending differential data instead of the pure sensed data.

However, due to the frequent changes in the positions of the nodes, which may imply false data transmissions and alter the encryption result, we assume that SRDA is not recommended for mobile WSNs. In addition, SRDA underexploits the high resource capacity of the heterogeneous sensor nodes as the used security mechanism is simple and does not guarantee a high security level. The introduced computation delay generated by the symmetric encryption may also reduce the data aggregation accuracy and compromise the data aggregation process.

### 2.3. Two Levels Secure Data Aggregation Protocols

**Figure 5:**
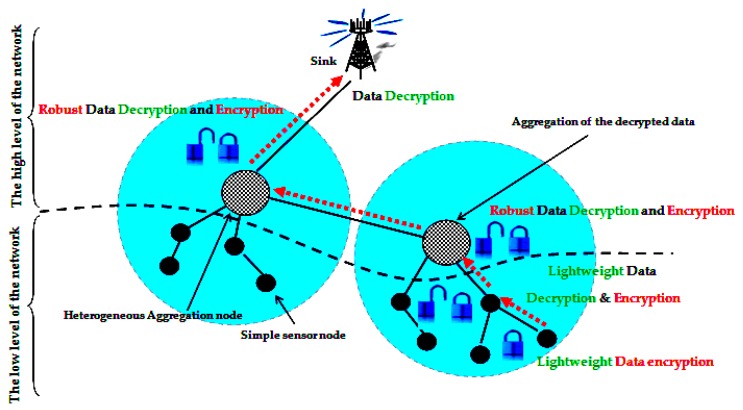
Two levels data aggregation security approach.

In the two levels data aggregation security protocols, the network is divided into two parts based on the sensor nodes resource capacities. As shown in Figure 5, the low level of the network contains homogeneous sensor nodes with limited resource while the high level of the network is formed by heterogeneous high resource capacity sensor nodes. According to the resource constraints of the low level of the network, the data aggregation security mechanism is supposed to be lightweight and use simple security algorithms. On the other side, the high level of the network can tolerate more complex and robust security algorithms to exploit the high resource capacity of the heterogeneous nodes. The two levels data aggregation security approach has been recently introduced in [22] where the authors proposed a data aggregation security algorithm for each network level.

#### 2.3.1. A Watermarking-Based Mechanism for Data Aggregation Security in Heterogeneous WSNs

In [22,23] the authors proposed a two level data aggregation security protocol which applies the watermarking concept and exploits the benefits of the cross layer architecture approach. The main idea of this protocol is to apply an efficient lightweight security scheme on resource limited homogeneous simple sensor nodes, while the heterogeneous node capacity is exploited by using a high security level mechanism without restricting the use of data aggregation functions. Two algorithms have been proposed at each network level to ensure data aggregation security according to the sensor nodes resource capacities. Indeed the Secure Data Aggregation Watermarking-based Scheme in Homogeneous WSNs algorithm (SDAW) was proposed at the low level of the network and a Cross-layer Watermarking-based Mechanism for Data Aggregation Integrity in Heterogeneous WSNs algorithm (CLDWA) was introduced at the high level of the network.

##### Secure Data Aggregation Watermarking-Based Scheme in Homogeneous WSNs (SDAW)

Most of the security protocols, used in data aggregation, aim to secure the network at the level of the aggregation nodes and to establish a secure channel between the aggregation nodes and the base station. However, the collected data, which represent an essential part of the aggregation process, come from a lower level; namely the homogeneous simple sensor nodes. Therefore, securing this level and the collected data becomes a good research option. Most of the studied protocols are interested in securing the higher levels. They also propose encryption mechanisms, which are mostly symmetrical, to secure the low level. This option certainly provides some degree of security, but imposes the computation overhead due to the keys’ distribution mechanisms. Indeed, the sensor nodes have very limited capacities. Therefore, they cannot tolerate the keys’ generation and storage mechanisms. In addition, the key distribution mechanism used between these nodes and the heterogeneous nodes implies additional security problems.

**Figure 6:**
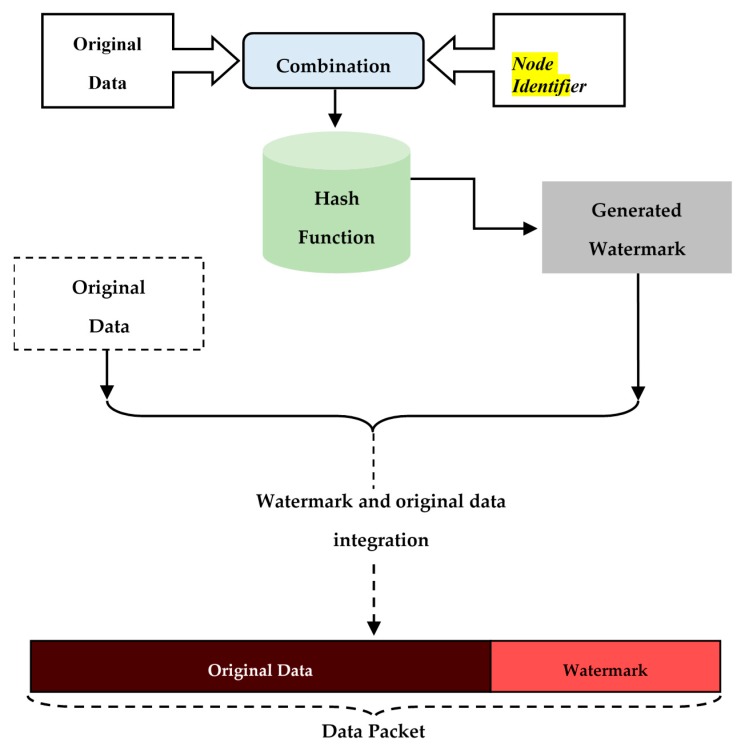
SDAW embedding (the sensor nodes level).

In this context, we have proposed in a previous work a new secure data aggregation protocol named Secure Data Aggregation Watermarking-based Scheme in Homogeneous WSNs (SDAW) [23], which represents a watermark based mechanism to secure data aggregation in the low level of the network. In the proposed mechanism, the watermark is generated using the collected data which is combined with the identifier of the node using the simple XOR function. Then, the one way hash function SHA-1 [24] is applied to the obtained result to generate the watermark. The watermark is then concealed in the data packet so that it will represent an integrated part of the original data which is also inserted into the data packet. Figure 6 presents the SDAW embedding algorithm at the sensor nodes level.

**Figure 7:**
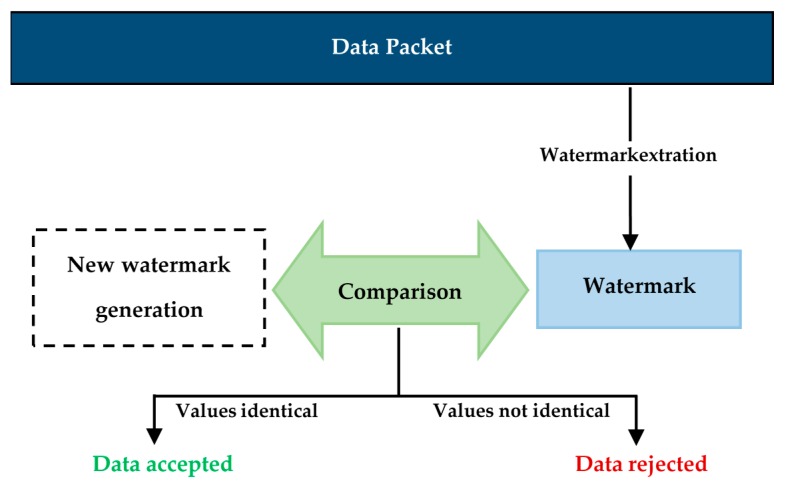
SDAWextraction and verification (the sensor nodes level).

When the data packet is received, the receiver node will extract the watermark. Then the node will use the original data contained in the data packet to generate a new watermark. The values of the extracted and the newly generated watermarks are compared. If they are identical, the node will accept the data and if they are not identical, the node will suppose that the data has been altered and will reject it. The proposed detection and verification mechanism is represented in Figure 7.

Compared to the other proposed mechanisms, SDAW offers a good security level while respecting the limited capacities of the sensor nodes. Indeed, SDAW uses a lightweight but reliable security primitive. In addition, contrary to the proposed solutions based on symmetric encryption, which involves additional computation to generate and distribute the keys, and which requires more memory to store the keys, our proposed mechanism SDAW does not encrypt data but is based on the watermark dissimilation which makes it difficult to differentiate the watermark from the original data. Thus, the energy and the memory of the sensor nodes are conserved.

To demonstrate the performance of SDAW, it was compared to another hop by hop security mechanism named SecureDAV [25]. SecureDAV is one of the most energy efficient protocols based on the use of the elliptic curves which offer a high security level while respecting the sensor nodes’ limited resources. Several evaluation metrics have been used in the simulation analyses such as the communication overhead, the false positive detections rate, the average delay, the data aggregation accuracy, and the total energy consumption. The obtained results demonstrate that SecureDAV protocol introduces much higher delay than SDAW protocol in the data aggregation process. The introduced delay is about 13.5 s in SecureDAV and is about 46.71% less in SDAW when 100% of the network sensor nodes are alive. Also, the simulation results show that SDAW achieves about 17% more data aggregation accuracy than SecureDAV when the introduced average delay is in its higher level and achieves about 39% more data aggregation accuracy than SecureDAV when the data aggregation accuracy reaches its minimal level. This confirms that SDAW offers a high degree of data aggregation accuracy compared to SecureDAV. SDAW is energy efficient and introduces very low energy consumption compared to SecureDAV. The simulation experiments show that SDAW consumes about 16.2 J (8.1% of the total network energy (200 J)) compared to SecureDAV which consumes 36.45% (72.9 J) of the total network energy.

##### Cross-Layer Watermarking-Based Mechanism for Data Aggregation Integrity in Heterogeneous WSNs algorithm (CLDWA)

**Figure 8:**
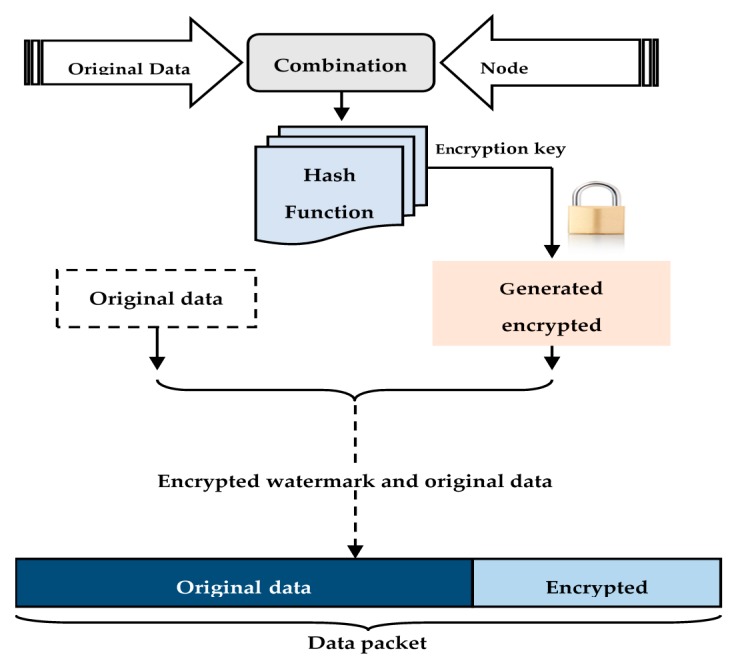
CLDWA embedding (the heterogeneous nodes level).

At the high level of the network CLDWA was proposed to secure the data aggregation process based on a cross-layer reinforced watermarking algorithm [22]. The fragile watermarking mechanism introduced by SDAW algorithm is reinforced with asymmetric encryption, where keys are used to generate and extract the watermark. Thus, the security between the heterogeneous nodes and the base station is enhanced while optimally exploiting the heterogeneous nodes higher capacities. In addition, unlike the privacy homomorphism encryption which offers restricted aggregation functions (only addition and multiplication functions are allowed), the proposed CLWDA algorithm does not apply any restrictions on the data aggregation functions. CLDWA embedding mechanism is represented in Figure 8.

Like the SDAW algorithm, the watermark generation in CLWDA is simple and uses a XOR function to combine the sensed data and the MAC address of the sensor. MD5 message digest algorithm is then used to hash the obtained result. The fragile watermark generation mechanism is enhanced with asymmetric encryption. Thus, the base station and the heterogeneous nodes generate and exchange the encryption keys, using an Elliptic Curve Diffie-Hellman algorithm (ECDH) [26], to encrypt the generated watermark.

**Figure 9:**
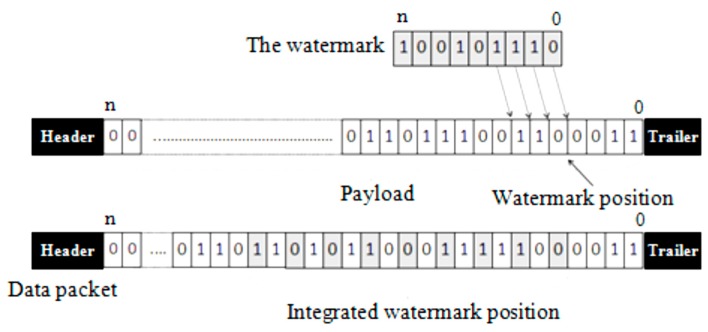
The watermark integration process.

The integration of the watermark into the data packet is based on a Cross-layer watermark embedding mechanism through which the watermark position is dynamically attributed and varies frequently. For this, CLWDA uses the wake-up time of the sensor nodes to compute the watermark position on the data packets. Therefore, each sensor node uses the wake-up time of the neighbour nodes to calculate and identify the watermark position in the data packet. Figure 9 presents the integration process.

**Figure 10:**
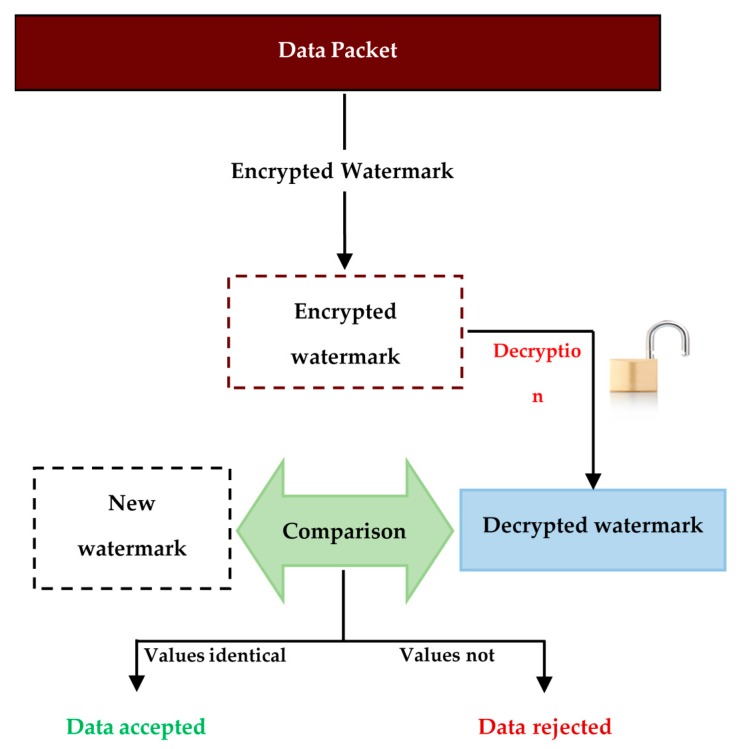
CLDWA extraction and verification (the heterogeneous nodes level).

The detection and verification mechanism of CLWDA algorithm operates practically like the SDAW algorithm. When the data packet is received, the heterogeneous node or the base station will extract the watermark and decrypt it according to the cross-layer watermark extraction mechanism. Then, the node will use the original data contained in the data packet to generate a new watermark. The values of the extracted and the newly generated watermarks are compared. If they are identical, the node will accept the data and will reject it otherwise. The proposed detection and verification mechanism in the high level of the network is represented in Figure 10.

The performances of CLWDA have been analyzed and compared to another heterogeneous and end to end based security mechanism named CDAP [7]. The obtained results clearly note that CLWDA consumes reasonable memory space compared to CDAP protocol. Thus, it can be supported by most of the lowest resource sensor nodes platforms (such as MICA2 and TelosB). The energy consumption of CLWDA was also analyzed and compared to CDAP and the simulation results show that CLWDA is energy efficient and consumes 91.58% less energy compared to CDAP. In addition, CLWDA protocol consumes seven times less energy than CDAP protocol, which demonstrates the energy efficiency of the reinforced fragile watermarking mechanism. On the other side, CLWDA reduces the computation delay introduced by 86.97% compared to CDAP protocol.

To demonstrate the performance of CLWDA protocol compared to CDAP protocol in terms of data aggregation, several simulation experiments have been conducted where four different aggregation functions are used. The first aggregation function (F1) is simple and computes the average of the received data. The second aggregation function (F2) is more complex and computes the average of the collected data only if the value of all received data packets is different from a fixed threshold such as temperature. The third aggregation function (F3) is also complex and computes the difference between the received sensed values if they are greater than a fixed threshold. The last aggregation function (F4) computes the variance value of the received data on the aggregation nodes. The simulation results demonstrated that CDAP, like most of the homomorphism based encryption algorithms, can only use addition and multiplication functions to aggregate the encrypted data. Therefore, the use of complex data aggregation functions is not allowed, which may affect the data aggregation process and reduce the data aggregation accuracy. On the other hand, the watermarking concept does not restrict the use of the aggregation functions, which optimizes the data aggregation.

##### Evaluation

Based on the experimental simulation results we can assume that the proposed two levels based data aggregation security protocol offers better performances compared to privacy homomorphism based protocols. Indeed, the watermarking based security scheme dose not restrict the use of the data aggregation function which optimizes the data aggregation degree. Also the reinforced watermarking algorithm exploits the high capacity of the heterogeneous sensor nodes and offers a good security level. The use of the lightweight fragile watermark at the level of the limited sensor node capacity optimizes data aggregation accuracy and the energy efficiency of the network. However, the watermarking based security scheme does not provide all the security properties (such as the data confidentiality) and was designed only to guarantee data integrity and authentication, which represents a security breach.

## 3. Heterogeneous Data Aggregation Security Approaches Evaluation and Discussion

Heterogeneous WSNs have opened a new research direction for several research problems, especially in the security domain. Securing the data aggregation process is one of the security problems which have been widely addressed in homogeneous WSNs, contrary to the heterogeneous WSNs where only a few works have addressed the issue. Based on the used data aggregation security approach, we have classified the existing heterogeneous data aggregation security protocols into three categories: End to end, hop by hop and two levels data aggregation security protocols. The end to end data aggregation security is the most popular approach since it was adopted by the majority of the proposed data aggregation security protocols. The main idea is to use the privacy homomorphism algorithms through which the aggregation functions can be applied directly to the encrypted data, thus protecting the integrity of the data while transmitting it from the aggregation nodes to the base station.

The end to end data aggregation security protocols offer a high security level and exploit the high resource capacities of the sensor nodes. However, the use of the homomorphism based algorithms introduces several drawbacks. Indeed, the use of data aggregation functions is restricted to only multiplication and addition functions. In addition, privacy homomorphism based protocols suppose that non heterogeneous nodes (classical sensor nodes with low resource capacities) are charged to encrypt the sensed data based on symmetric key and send them to the aggregation nodes (heterogeneous nodes), which are charged to aggregate the received data using the privacy homomorphism algorithm. However, the encryption mechanism can bring extra charges for resource limited non heterogeneous sensor nodes. Table 1 summarizes the advantages and limits of each studied end to end based data aggregation security protocol. Table 2 defines the security primitives used by each protocol.

The good security level offered by the end to end data aggregation security protocols does not mean that all main security issues have been ensured (data confidentiality, data integrity, data freshness and authentication). Indeed, authentication is ensured only by PIA and RCDA protocols where data freshness is ensured by SDKAM and Sen-SDA protocols. On the other hand, data integrity is not ensured by CDAP protocol. Table 3 presents the ensured security issues of each end to end data aggregation security protocol.

Contrary to the end to end data aggregation security approaches, the hop by hop data aggregation security approach encrypts and decrypts data at each aggregation node to provide strong data aggregation (no restriction on the use of the data aggregation functions) while ensuring data integrity and authentication. However, the data confidentiality cannot be ensured and the relayed data is thus more vulnerable to malicious attacks. Also, the hop by hop data aggregation security approach supposes that all the network nodes apply high computation cryptographic algorithms which is inefficient for the resource limited homogeneous sensor nodes deployed in the network. For these reasons, the hop by hop based data aggregation security protocols are not largely used, particularly when security is related to sensitive application areas.

The third data aggregation security approach tries to overcome the limits of the two previous approaches by optimizing the data aggregation process, exploiting the high capacity of the heterogeneous aggregation nodes and respecting the limitation of the simple homogeneous sensor nodes. The main idea is to divide the network based on the sensor nodes’ capacities to efficiently balance the work load through the network. Consequently, the high computational data aggregation security algorithms are applied to the heterogeneous aggregation nodes to secure the data aggregation process while simple and lightweight security mechanisms are applied to resource constrained nodes to guarantee a secure data collection. The two levels data aggregation security approach remains a fresh research area and was recently introduced in [22,25] where the SDAW and CLWDA protocols were proposed to secure the low and the high level of the network, respectively. Despite the several performance features introduced by the SDAW and CLWDA methods in terms of data aggregation optimization and energy efficient security, the proposed watermarking-based approach does not cover all the security issues.

## 4. Conclusions

In this paper, we have addressed the problem of data aggregation security in heterogeneous wireless sensor networks, pointing out the needs, the constraints and various defense mechanisms used to solve the security problems. Indeed, heterogeneous WSNs are basically designed to address the limitations of conventional homogeneous WSNs. Heterogeneous nodes are supposed to have large capabilities which widely exceed those of the conventional nodes. Hence, introducing high data aggregation security algorithms which are cannot be used by classic nodes, and which offer the possibility of using many aggregation functions, is beneficial. Several complex security primitives can also be combined. The large storage space of the heterogeneous nodes can also be exploited to contain a large number of keys (for random key generation), increasing the security degree. The data aggregation security problem in heterogeneous WSNs remains a new research area and we have tried in this paper to shed light on it by presenting a survey of the few proposed protocols. We have also proposed a new classification of the surveyed protocols, based on the adopted data aggregation security strategy. Finally, an evaluative discussion is given at the end of the paper to analyze the advantages and the disadvantages of each heterogeneous data aggregation security category.

## Figures and Tables

**Table 1:** The end to end heterogeneous data aggregation security protocols.

Protocol	Advantages	Limits
CDAP	- Improves the security between the base station and the AGGNODEs.	- Only addition and multiplication aggregation functions are allowed. - Computation overhead on resource limited sensor nodes
PIA	- A hybrid protocol combining different solutions to increase security.	- Due to privacy homomorphism and OPES, the protocol is confronted to the limited allowed aggregation function.
SEDA	- Improves security by offering a random key generation.	- Improves security by offering a random key generation.
SDKAM	- Uses an effective keys management system to strength the security.	- Encryption IHC is limited in terms of allowed aggregation functions.
RCDA	- Uses digital signatures to guarantee data integrity and authentication. - The base station has the possibility to recover all sensed data even if they were already aggregated.	- The network is not scalable. - The key generation and distribution mechanisms are not presented.
Sen-SDA	- Insures end-to-end confidentiality. - Insures hop by hop authentication. - Reduces the computational cost through the use of efficient pairing free identity based signature scheme.	- The homomorphic encryption limits the aggregation functions to only addition and multiplication. - Computation overhead on resource limited sensor nodes due to the use of asymmetric encryption.

**Table 2:** Security primitives of the heterogeneous end to end data aggregation security protocols.

	MAC Authentication	Symmetric Encryption	Asymmetric Encryption	Privacy Homomorphism
CDAP	No	RC5	Okamoto and Uchiyama public-key cryptosystem	Okamoto and Uchiyama public-key cryptosystem
PIA	MAC message	Order Preserving Encryption Scheme OPES	No	SHIA SUM algorithm
SEDA	No	One-time pad method	No	No
SDKAM	No	Yes	Yes	IHC
RCDA	MAC message	KeyGen of Boneh *et al.*’s scheme KeyGen of Mykletun *et al.*’s scheme	No	No
Sen-SDA	No	No	EC-El Gamal^+^	EC-El Gamal^+^

**Table 3:** Ensured security issues of end to end data aggregation security protocols.

	Confidentiality	Integrity	Freshness	Authentication
CDAP	Yes	No	No	No
PIA	Yes	Yes	No	Yes
SEDA	Yes	Yes	No	No
SDKAM	Yes	Yes	Yes	No
RCDA	Yes	Yes	No	Yes
Sen-SDA	Yes	Yes	Yes	No

## References

[1] Simmons G.J. (1979). Symmetric and Asymmetric Encryption. ACM Comput. Surv. CSUR.

[2] Jueneman R.R., Matyas S.M., Meyer C.H. (1985). Message Authentication. IEEE Commun. Mag..

[3] Rivest R.L., Adleman L., Dertouzos M.L. (1978). On data banks and privacy homomorphisms. Found. Secur. Comput..

[4] Yu L., Wang N., Zhang W., Zheng C. Deploying A Heterogeneous Wireless Sensor Network. Proceedings of the International Conference on Wireless Communications, Networking and Mobile Computing.

[5] Mlaih E., Aly S.A. Secure Hop-by-Hop Aggregation of End-to-End Concealed Data in Wireless Sensor Networks. Proceedings of the IEEE INFOCOM Workshops.

[6] Josna J., Joyce J., Fijo J. (2012). A Survey on Secure Data Aggregation Protocols in Wireless Sensor Networks. Int. J. Comput. Appl..

[7] Ozdemir S. Concealed data aggregation in heterogeneous sensor networks using privacy homomorphism. Proceedings of the IEEE International Conference on Pervasive Services.

[8] Rivest R.L. The RC5 Encryption Algorithm. Proceeding of the Second International Workshop.

[9] Taban G., Gligor V.D. Privacy-preserving integrity-assured data aggregation in sensor networks. Proceeding of International Symposium on Secure Computing.

[10] Bellare M., Canetti R., Krawczyk H. Keying hash functions for message authentication. Proceedings of Annual International Cryptology Conference.

[11] Agrawal R., Kiernan J., Srikant R., Xu Y. Order preserving encryption for numeric data. Proceedings of the 2004 ACM SIGMOD International Conference on Management of Data.

[12] Chan H., Perrig A., Song D. Secure hierarchical in-network aggregation in sensor networks. Proceedings of the 13th ACM Conference on Computer and Communications Security.

[13] Huang S.I., Shieh S., Tygar J.D. (2010). Secure encrypted-data aggregation for wireless sensor networks. Wirel. Netw..

[14] Lloret J., Garcia M., Tomas J., Boronat F. (2008). GBP-WAHSN: A Group-Based Protocol for Large Wireless Ad Hoc and Sensor Networks. J. Comput. Sci. Technol..

[15] Sandhya M.K., Murugan K. Secure Framework for Data Centric Heterogeneous Wireless Sensor Networks. Proceeding of the Recent Trends in Network Security and Applications—Third International Conference, CNSA 2010.

[16] Chan A.C.-F. Symmetric-Key Homomorphic Encryption for Encrypted Data Processing. Proceeding of the IEEE International Conference ICC’09.

[17] Chen C.M., Lin Y.H., Lin Y.C., Sun H.M. (2012). RCDA: Recoverable concealed data aggregation for data integrity in wireless sensor networks. IEEE Trans Parallel Distrib. Syst..

[18] Kyung S., Cheol P. (2014). A secure data aggregation scheme based on appropriate cryptographic primitives in heterogeneous wireless sensor networks. IEEE Trans. Parallel Distrib. Syst..

[19] Law L., Matt B.J. Finding invalid signatures in pairingbasedbatches. Proceedings of the 11th IMA International Conference.

[20] Sanli H.O., Ozdemir S., Cam H. SRDA: Secure reference-based data aggregation protocol for wireless sensor networks. Proceeding of the 60th IEEE Vehicular Technology Conference.

[21] Rivest R.L., Robshaw M.J.B., Sidney R., Yin Y.L. (1998). The RC6 Block Cipher, AES Submission. http://theory.lcs.mit.edu/rivest/rc6.pdf.

[22] Boubiche D.E., Boubiche S., Bilami A. (2015). A Cross-layer Watermarking-based Mechanism for Data Aggregation Integrity in Heterogeneous WSNs. IEEE Commun. Lett..

[23] Boubiche D.E., Boubiche S., Homero T., Al-Sakib K.P., Bilami A., Athmani S. (2015). SDAW: Secure Data Aggregation Watermarking-Based Scheme in Homogeneous WSNs. J. Telecommun. Syst..

[24] Eastlake D., Jones P. (2001). RFC3174-US Secure Hash Algorithm-I (SHA1); Network Working Group. http://www.hjp.at/doc/rfc/rfc3174.html.

[25] Mahimkar A., Rappaport T.S. SecureDAV: A Secure Data Aggregation and Verification Protocol for Sensor Networks. Proceedings of the IEEE Global Telecommunications Conference.

[26] Wang S., Cao Z., Strangio M.A., Wang L. (2008). Cryptanalysis and improvement of an elliptic curve Diffie-Hellman key agreement protocol. IEEE Commun. Lett..

